# *Staphylococcus aureus* Alpha-Toxin Induces the Formation of Dynamic Tubules Labeled with LC3 within Host Cells in a Rab7 and Rab1b-Dependent Manner

**DOI:** 10.3389/fcimb.2017.00431

**Published:** 2017-10-04

**Authors:** María M. López de Armentia, María C. Gauron, María I. Colombo

**Affiliations:** Laboratorio de Biología Celular y Molecular, Instituto de Histología y Embriología, Facultad de Ciencias Médicas, CONICET, Universidad Nacional de Cuyo, Mendoza, Argentina

**Keywords:** *Staphylococcus aureus*, autophagy, bacterial pathogens, intracellular bacteria, bacteria-induced tubules

## Abstract

*Staphylococcus aureus* is a pathogen that causes severe infectious diseases that eventually lead to septic and toxic shock. *S. aureus* infection is characterized by the production of virulence factors, including enzymes and toxins. After internalization *S. aureus* resides in a phagosome labeled with Rab7 protein. Here, we show that *S. aureus* generates tubular structures marked with the small GTPases Rab1b and Rab7 and by the autophagic protein LC3 at early times post-infection. As shown by live cell imaging these tubular structures are highly dynamic, extend, branch and grow in length. We have named them *S*. *a**ureus* induced filaments (Saf). Furthermore, we demonstrate that the formation of these filaments depends on the integrity of microtubules and the activity of the motor protein Kinesin-1 (Kif5B) and the Rab-interacting lysosomal protein (RILP). Our group has previously reported that α-hemolysin, a secreted toxin of *S. aureus*, is responsible of the activation of the autophagic pathway induced by the bacteria. In the present report, we demonstrate that the autophagic protein LC3 is recruited to the membrane of *S. aureus* induced filaments and that α-hemolysin is the toxin that induces Saf formation. Interestingly, increasing the levels of intracellular cAMP significantly inhibited Saf biogenesis. Remarkably in this report we show the formation of tubular structures that emerge from the *S. aureus*-containing phagosome and that these tubules generation seems to be required for efficient bacteria replication.

## Introduction

*Staphylococcus aureus* is a leading agent of severe bacterial infections. It may cause diseases, such as endocarditis, osteomyelitis, pneumonia and meningitis. This pathogen has an important capacity to invade the vascular system from local infection sites and to disseminate passing across the endothelial barrier, leading to bacteremia and sepsis.

Once the bacterium reaches the bloodstream it is phagocytosed by polymorphonuclear neutrophils (PMN) and macrophages. Recent publications have shown that *S. aureus* also efficiently invade non-professional phagocytes, such as epithelial and endothelial cells, fibroblasts, osteoblasts and keratinocytes leading to host cell death. *S. aureus* phagocytosis by non-professional cells is mediated by a zipper-type mechanism including integrins and adhesins (Fowler et al., 2000; Kintarak et al., 2004; Edwards et al., 2011). Once internalized, it transits the phagosomal pathway avoiding lysosomal degradation to finally scape from phagosomes in a toxin-dependent mechanism, further replicating in the cytoplasm (Grosz et al., 2014). Kurt and colleagues showed that *S. aureus* can localize into autophagosomes but their maturation is blocked and the fusion with lysosomes is inhibited, allowing bacterial replication. Afterwards, the bacteria induce apoptosis through a caspase-independent mechanism. Interestingly, *S. aureus* strains deficient for *agr*, a global regulator of *S. aureus* virulence, were not targeted by autophagy and did not cause host-cell death (Schnaith et al., 2007). We have previously shown that autophagy induction in infected cells is mediated by the staphylococcal-toxin α-hemolysin (Hla), a pore forming protein secreted as a water soluble monomer capable to bind and oligomerize on the host cell membrane (Mestre et al., 2010; Berube and Wardenburg, 2013). When cells are exposed to the Hla purified toxin there is an increased accumulation of vesicles labeled with LC3, that have characteristics of non-acidic and non-degradative compartments, suggesting that the maturation of these autophagic structures is blocked (Mestre et al., 2010). In addition, the toxin secreted by the internalized bacteria also stimulated autophagy, as cells infected with the wild-type strain of *S. aureus* showed recruitment of LC3 to the phagosomal membrane but did not accumulate lysotracker, dye that stains acidic compartments. In contrast, those phagosomes containing *S. aureus* strain Hla (–), which is unable to produce the toxin, were include in an acidic compartment unlabeled by LC3 (Mestre et al., 2010).

In the last few years there has been many studies focusing on the molecules involved in the autophagic pathway and genetic studies in yeast have led to the discovery of several Atg (autophagy related) genes, many of which have mammalian orthologs (Füllgrabe et al., 2016). ULK1 (unc-51 like autophagy activating kinase 1) activates the lipid kinase VPS34, stimulating the synthesis of phosphatidylinositol 3-phosphate (PI3P) and the formation of an omegasome, at the region were Atg9 vesicles align with the ER (Karanasios et al., 2016). Atg5 interacts with Atg12 (Atg5-Atg12 complex) covalently and non-covalently with Atg16. The microtubule-associated protein-1 light chain-3 (MAP1-LC3/Atg8/LC3) is cleaved by Atg4 to form a soluble protein that localizes into cytoplasm termed LC3-I. Then, LC3-I is lipidated to generate LC3-II which is capable of binding to membranes. LC3-II is formed at the place where the Atg12-Atg5-Atg16 complex is localized and is able to associate with autophagosomal membranes, even when autophagosomes fuse with lysosomes to form autolysosomes (Rubinsztein et al., 2009).

Autophagy is classically regulated by two important proteins; one is the phosphatidylinositol-3-kinase (PI3K) Class III, which activates the autophagic pathway. The kinase Class III PI3K and its human ortholog hVps34 interact with p150 myristoylated kinase and Beclin-1 to activate Atg proteins. The other one is the serine/threonine kinase mTOR (mechanistic target of rapamycin), a sensor of cellular energy and amino acid levels, which inhibits autophagy (Gallagher et al., 2016). However, the autophagic response induced by *S. aureus* is atypical and involves cAMP, EPAC (exchange protein activated by cAMP) and the small GTPase Rap2b, excluding the canonical pathway of PI3K/Beclin-1. We have previously demonstrated that incubation of cells with dbcAMP and subsequent infection with *S. aureus* inhibit the autophagy activation induced by the bacterium, but the cAMP-dependent protein kinase A (PKA) was not involved in the cAMP inhibition of Hla induced autophagy. Indeed, we have shown that this regulation mechanism involves EPAC (exchange protein activated by cAMP) and Rap2b that negatively regulate the autophagic response induced by the toxin (Mestre and Colombo, 2012).

Despite these evidences, the events that lead to *S. aureus* replication avoiding its destruction are not clear yet. Here, we report the formation of dynamic tubular structures induced by *S. aureus*, which protrude from the bacterial-containing phagosome toward the periphery of the cell. The *S. aureus*-induced filaments (Safs) are labeled by host cell proteins, such as Rab7, Rab1b and the autophagic protein LC3, which are essential for Saf formation. Additionally, we demonstrate that Safs elongation requires microtubules and kinesin1. Remarkably, the formation of Safs is dependent on α-toxin-competent bacteria and also they are induced by purified α-hemolysin. In summary, we are showing that *S. aureus* induces filamentous structures which are critical for efficient bacterial replication.

## Materials and methods

### Materials

α-MEM cell culture medium was purchased from Gibco (Invitrogen, 11965-175) and fetal bovine serum (A15-101) from PAA (GE Healthcare Argentina S.A., Argentina). Nocodazole, Chloramphenicol, Gentamicin, Wortmannin and α-hemolysin from *Staphylococcus aureus* (H9395) were obtained from Sigma Aldrich (Buenos Aires, Argentina). Geneticin (G418) was purchased from Gibco (Invitrogen, Argentina).

### Plasmids

The pEGFP-LC3, pRFP-LC3, pEGFP-Rab1b, pEGFP-Rab11, and pEGFP-Rab24 constructs were obtained as described in earlier publications (Munafó and Colombo, 2002; Savina et al., 2005; Zoppino et al., 2010). The plasmid pEGFP- Rab7wt was a generous gift from Bo van Deurs (University of Copenhagen) and the plasmid encoding enhanced GFP (EGFP)-Rab5wt was kindly provided by Dr. Philip D. Stahl (Washington University). The pCDNA Kif5wt, pCDNA Kif5T93N were kindly provided by Dr. Alfredo Cáceres (Mercedes and Martín Ferreyra Institute, Córdoba, Argentina). The pRFP-RILP and mCherry-RILP ΔN was gently provided by Dr. Walter Berón (IHEM, Mendoza, Argentina). The pEGFP-Rab34 was gently provided by Dr. Maximiliano Gutiérrez (Helmholtz Centre for Infection Research, Braunschweig, Germany). The pEGFP-Rab29 and pEYFP-Rab32 were gently provided by Drs. Jorge Galán and Stefania Spano (Yale University School of Medicine, New Haven).

### Cell culture and transfection

CHO-K1 (ATCC) were grown in α-MEM supplemented with 10% FBS, streptomycin (50 μg/ml) and penicillin (50 U/ml). Stably transfected CHO cells overexpressing pEGFP-LC3, pEGFP-Rab1b or pEGFP-Rab7 wild type and pEGFP-Rab7T22N were selected with 0.5 mg/ml of geneticin. Cells were transfected or cotransfected using Lipofectamine 2000 (Invitrogen), according to the manufacturer's instructions.

### Bacterial strains and growth conditions

*Staphylococcus aureus* strain wild type (8325-4), the mutant deficient for α-hemolysin Hla (–) (DU1090) or the Hla (–) mutant complemented with a α-hemolysin plasmid (DU1090/pDU1212) [Hla(+)] were kindly provided by Dr. Claudia Sola (CBICI-CONICET, Córdoba, Argentina). These bacterial strains were grown overnight at 37°C in 10 ml of Luria-Bertani (LB) broth with appropriate antibiotics. Bacteria were resuspended in infection medium (αMEM containing 10% FBS) at an OD_650_ of 0.4 (approximately 4 × 10^8^ CFU) and then were diluted to achieve a multiplicity of infection (MOI) of 10:1 (bacteria:cell) in the infection medium. After 1 h of infection, the medium was washed to remove extracellular bacteria and new infection medium was added to cells. Subsequently, cells were incubated for different periods of times (2, 4, or 6 h) and fixed with 4% paraformaldehyde (PFA) or processed as *living cells* by confocal microscopy. In all cases, after 1 h of infection cells were washed and gentamicin (100 μg/ml) was added for 30 min to kill extracellular bacteria.

### Bacteria labeling

For analysis in fixed samples, bacterial DNA was labeled with 45 nM Hoechst in Mowiol. For live imaging, bacteria were incubated with Rhodamine-Red (5 μg/ml) for 1 h at 37°C, subsequently washed with PBS twice and then added to the cells. In some live experiments Hoechst or TOPRO was added to the infection medium and visualized directly on the microscopy chamber.

### Obtaining *S. aureus* supernatant

*Staphylococcus aureus* strains wt, the mutant deficient for α-hemolysin Hla(–) or the Hla(+) mutant complemented with an α-hemolysin plasmid were grown at 37°C with shaking in 10 ml of LB broth with appropriate antibiotics to an optical density at 600 nm (OD600) of 2.0. After removal of the bacterial cells by centrifugation at 12,000 g for 15 min at 4°C, the obtained supernatants were filtered using a 0.22 mm Millipore filter. The resulting filtrate was used as supernatant for experiments. Supernatants were checked for absence of bacteria by cultivating small aliquots on LB agar. Protein concentration was determined by the Bradford assay.

### Treatment with the toxin

CHO GFP-LC3 cells were incubated for 1 h with 10 μg/ml of purified α-hemolysin from *S. aureus* (Sigma). Live cells were analyzed by fluorescence microscopy.

### Fluorescence microscopy and imaging

CHO cells were analyzed by fluorescence microscopy using an Olympus Confocal FV1000. The program FV10-ASW 3.0 was used for all acquisitions and settings for the live imaging. For spinning disk acquisition, we used a Quorum Technologies WaveFX Confocal Microscope with a Yokogawa CSU-X1 Spinning Disc Unit and a LCI Temperature Controller and CO_2_ Mixer for Live Cell Imaging (Neurobiology of Stress CNS, University of Toronto at Scarborough, Canada). The resulting movies series were corrected to improve the contrast and resolution using the deconvolution plugin on ImageJ. The image analysis of Saf growth and contraction was carried out using ImageJ.

### Colocalization analysis

For colocalization studies images were processed using the plugin Colocalization analysis from ImageJ. Images were deconvolved and then analyzed using Mander's Overlap Coefficient (MOC) and Pearson Correlation Coefficient (PCC) to determine the degree of overlap between images using the green and red channel. Data are representative of two different experiment (*n* = 20 cells/condition).

### Live cell imaging

Cells were cultured in cover glasses of 25 mm of diameter and transfected when required. Infection with the *S. aureus* strains was performed as described above. After the proper infection time the medium was replaced by new infection medium and the cover glass was imaged at the required time-points after infection. The cover glasses were mounted on the microscope chamber equipped with a heating unit to maintain the temperature at 37°C.

### CFU determination

CHO cells were infected for 2 or 4 h with *S. aureus* wt. In some experiments CHO cells stably expressing EGPF-Rab7 or EGFP-Rab7T22N were used or CHO cells were transiently transfected with GFP-Rab1b wt or GFP-Rab1b S22N. In all cases, after 1 h of infection cells were washed and gentamicin (100 μg/ml) was added for 30 min to kill extracellular bacteria. After the infection period (i.e., 2 h) cells were washed with PBS and lysed with sterile double distilled water for 10 min at room temperature. Lysates were diluted with PBS, plated in Brain Heart agar and incubated for 12 h at 37°C. Colonies were counted on the plate with dilutions yielding 50–100 visible colonies.

## Results

### *S. aureus* induces formation of dynamic tubular structures decorated with Rab7 in CHO cells

**Figure 1:**
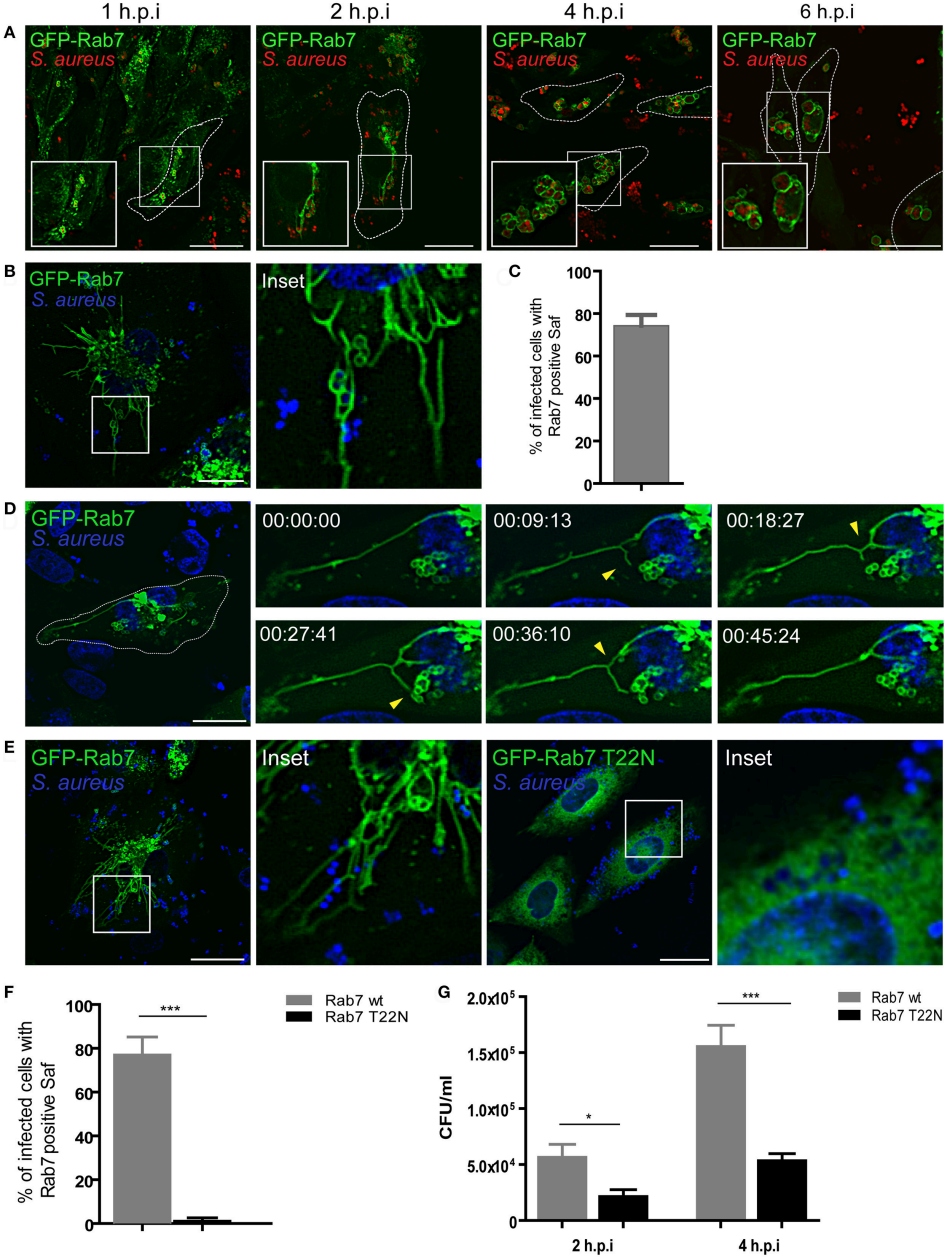
*S. aureus* induces formation of tubular structures decorated with Rab7. **(A)** GFP-Rab7wt stably transfected CHO cells were infected with *S. aureus* wt for 1 h (1 h.p.i) and then washed and incubated for additional 1 h (2 h.p.i), 3 h (4 h.p.i), or 5 h (6 h.p.i) to allow bacteria replication. Bacteria were labeled with rhodamine-red **(B)** GFP-Rab7 CHO cells were infected for 30 min with *S. aureus* and living cells were imaged. **(C)** Quantification of the percentage of infected cells that presented Saf formation. **(D)** Movie showing CHO cells overexpressing GFP-Rab7 and infected with *S. aureus*. Yellow arrow heads indicate Saf extension, branching and phagosome contact. Relative time-points are expressed as h:min:s. **(E)** CHO GFP-Rab7wt and CHO GFP-Rab7T22N (dominant negative mutant) stably transfected cells were infected for 1 h with *S. aureus* wt. Bacteria were labeled with Hoechst. **(F)** Quantification showing the percentage of cells with Rab7 positive Saf in cells overexpressing Rab7wt or Rab7 T22N. **(G)** The graph shows the quantification of the number of CFU/ml in CHO cells stably transfected with GFP-Rab7 (black bars) or GFP-Rab7T22N (gray bars) and infected with *S. aureus* wt for 2 or 4 h. In all cases data show mean ± SEM from three independent experiments (*n* = 150 cells/condition). ^*^P ≤ 0.01, ^***^P ≤ 0.0001 from two-tailed Student *t*-test. Scale bar: 20 μm.

It has been previously demonstrated that *S. aureus* reside in an unique phagosomal compartment that poorly localizes with LAMP but is highly decorated by Rab7 (Schnaith et al., 2007; Seto et al., 2011). Both publications present some contradictory observations regarding the staining with lysosomal markers however both conclude that the *S. aureus*-residing compartment acquires Rab7. In order to analyze the sequence of events that lead to the recruitment of Rab7 to the *S. aureus* phagosome, CHO cells stably overexpressing GFP-Rab7 were infected with *S. aureus* wild type (wt) and analyzed at four different post-infection times (1, 2, 4, and 6 h; Figure 1A). From early post-infection times the recruitment of Rab7 to the *S. aureus* containing-phagosome was observed. Interestingly, at these post-infection times we also observed tubular like structures that were marked with Rab7. At later post-infection times (4 h.p.i) these tubular structures were no longer visible. Instead, infected CHO cells presented *S. aureus* containing-phagosomes in a “honeycomb-like” distribution. Interestingly, the late endosomal/lysosomal protein LAMP-1 was recruited to *S. aureus* phagosome but no tubular structures labeled with LAMP-1 were observed at 1–2 h post-infection (Figure S1A).

In order to analyze the dynamics of these structures we observed living cells using confocal microscopy. For this purpose, CHO cells stably overexpressing GFP-Rab7 were infected with *S. aureus* wt and living cells were imaged with confocal microscopy in a temperature controlled chamber (Figure 1B and Movie S1). We observed the formation of highly dynamic tubules that extended from the *S. aureus* containing phagosome, thus we named these tubular structures *S*. *a**ureus*-induced filaments (Safs). Quantification analysis indicates that approximately 70% of the infected cells showed one or more Safs at 2 h.p.i. (Figure 1C). We next analyzed time-lapse series of living CHO cells stably transfected with GFP-Rab7 and infected with *S. aureus* wt and a representative cell is shown in Figure 1D (see also Movie S1). We noted the formation of tubular GFP-Rab7 positive structures extending from the *S. aureus* phagosomes toward the cell periphery as early as 1 h after infection (Figure 1D). The number and the extension of these tubular structures increased over time. Closer analyses of the time-lapse movies indicated that individual Safs were highly dynamic showing not only extension but also contraction, branching or contact with another *S. aureus*-containing vacuoles. Linear growth of Safs was observed as well as apparently random changes in the direction of Saf extensions. As a control, uninfected CHO GFP-Rab7 living cells were observed to assure that the formation of the tubules was restricted to the infected cells (Figure S1B).

Rab proteins are small GTPases regulated by specific guanine nucleotide exchange factors (GEFs), which catalyze the displacement of GDP and binding of GTP, by GTPase activating proteins (GAPs) which stimulate the GTP hydrolysis (Novick, 2016). We therefore studied the involvement of a functional Rab7 in Saf generation and elongation. For this purpose, CHO cells stably overexpressing an EGFP wild-type Rab7 or the dominant-negative mutant (Rab7-T22N) were infected with *S. aureus* wt (Figure 1E). After 1 h post-infection in Rab7 wt expressing cells we observed Saf formation; however, in cells overexpressing Rab7-T22N no Rab7 tubular structures were observed (Figure 1F), indicating that an active Rab7 is required for the formation of the filamentous structures.

With the purpose of determining the importance of a functional Rab7 on *S. aureus* replication, CHO cells stably overexpressing GFP-Rab7 wt or GFP-Rab7 T22N were infected with *S. aureus* wt. After 1 h post-infection cells were washed with PBS and gentamicin (100 μg/ml) was added to kill extracellular bacteria. Subsequently, cells were incubated an additional hour (2 h.p.i) or 3 h (4 h.p.i) and finally were lysed with sterile water and plated on Brain Heart Agar for colony forming units (CFU) determination. As shown in Figure 1G, cells overexpressing Rab7-T22N presented a significant decrease in bacterial replication compared to cells overexpressing Rab7 wt. These results indicate that Rab7 is essential for *S. aureus* replication and suggest that Saf formation is also important for efficient bacteria replication.

### Microtubules, kinesin-1 and RILP participate in saf elongation

**Figure 2:**
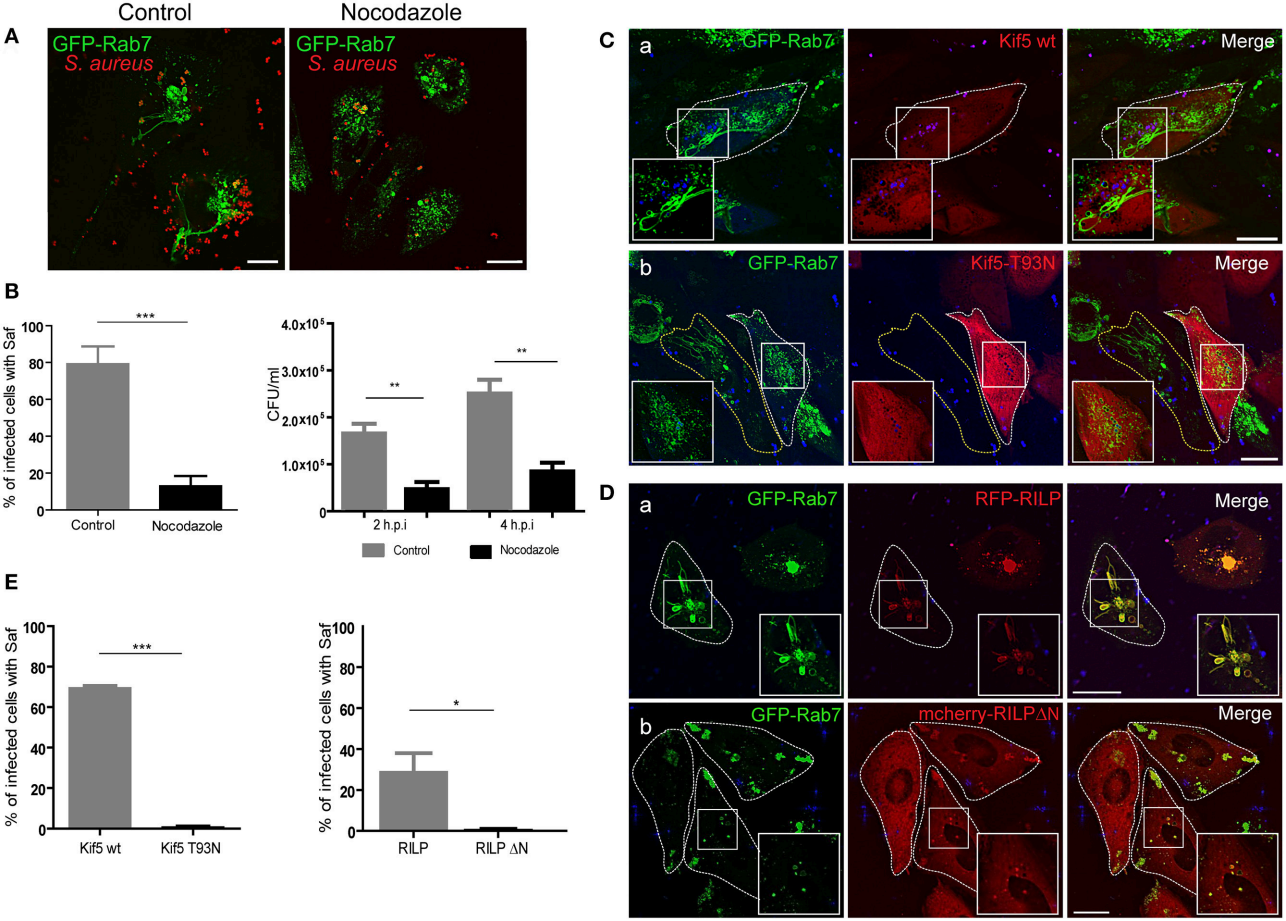
Microtubules, Kif5 and RILP are necessary for Saf elongation. **(A)** CHO GFP-Rab7 stably transfected cells were infected for 1 h with *S. aureus* wt. In the left panel cells were incubated in control media and in the right panel after 30 min post-infection 2 μM nocodazole was added and maintained for the rest of the infection period. **(B)** The graph on the left shows quantification of the percentage of infected cells with Saf formation in control conditions (gray bar) or treated with nocodazole (black bar). The graph on the right represents the quantification of the number of CFU/ml of CHO cells infected for 2 or 4 h *S. aureus* wt. Grey bars represent control conditions and black bars indicate cells treated with 2 μM nocodazole. **(C)** CHO cells stably overexpressing GFP-Rab7 were co-transfected with RFP vector and pcDNA-Kif5 wt (Panel a) or with pcDNA-Kif5T93N (panel b), and then subsequently infected with *S. aureus* wt for 1 h. **(D)** CHO GFP-Rab7 stably overexpressing cells were transfected with RFP-RILP wt (Panel a) or with mCherry-RILPΔN (Panel b) and then infected with *S. aureus* wt for 1 h. Bacteria were labeled with TOPRO. All conditions **(A,C,D)** were observed as living cells using confocal microscopy. Images are representative of two independent experiments. **(E)** Graphs show the percentage of cells with Rab7 and Kif5 double positive Safs and the percentage of cells with Safs marked with Rab7 an RILP. In all cases data show mean ± SEM of two independent experiments (*n* = 100 cells/condition). ^*^P ≤ 0.01, ^**^P≤0.001 ^***^P ≤ 0.0001 from two-tailed Student *t*-test. Scale bar: 20 μm.

The role of the GTPase Rab7 has been extensively investigated and it is well recognized to facilitate endosomal maturation, transport from the late endosome to the lysosome, and positioning of the endosomes and lysosomes by regulating their movement along the cytoskeleton (Wang et al., 2011). Nocodazole is a pharmacological inhibitor of microtubules (MT) that affects the cytoskeleton integrity. We tested the effects of nocodazole on the dynamic of Safs marked with Rab7 (Figure 2A); treatment with nocodazole did result in the loss of a large number of existing Safs (Figure 2B). It is important to take into account that cells were treated with nocodazole after 30 min of infection to guarantee a proper internalization of *S. aureus*, as microtubules and actin microfilaments are necessary for bacterial phagocytosis (Jevon et al., 1999; Shi and Zhang, 2012).

With the purpose of determining if the inhibition of Safs caused by nocodazole treatment affected bacteria replication we infected CHO cells with *S. aureus* wt for 30 min and then 2 μM of nocodazole was added. After 2 or 4 h post-infection cells were lysed and CFU was determined. As shown in Figure 2B a marked decrease in bacterial replication was observed in cells treated with the microtubule depolymerizing agent in contrast to control conditions (Figure 2B).

Microtubule-based motors include kinesins, which move toward the MT plus end, and dyneins that moves to the minus end (Akhmanova and Hammer, 2010). Kinesins are a family of molecular motors that use the energy of ATP hydrolysis to travel along the surface of microtubule filaments (Verhey and Hammond, 2009). In order to determine if Kinesin-1 is involved in Saf elongation, CHO cells stably expressing Rab7 were cotransfected with pcDNA-Kif5 wt or pcDNA-Kif5 T93N, a dominant negative mutant that has no ATPase activity. After protein expression cells were infected with *S. aureus* wt for 1 h and images from living cells were analyzed (Figure 2C). As shown in Figure 2C, when Kif5 wt was overexpressed we observed 70% of infected cells with Safs decorated with Rab7. In contrast, in cells overexpressing Kif5-T93N mutant Saf elongation was inhibited and no tubular structures marked with Rab7 were observed (Figure 2E). These results indicated that Saf formation and elongation requires kinesin-1 motor to extend toward the cellular periphery.

Rab7-interacting lysosomal protein (RILP) regulates microtubule minus-end directed transport by recruiting the dynein/dynactin motor complex (van der Kant et al., 2013). Thus, we transfected CHO cells stably overexpressing GFP-Rab7 with RFP-RILP wt or mCherry-RILPΔN, a truncated mutant with an N-terminal deletion that retains the ability to bind to active Rab7 but is unable to bind to microtubule motors (Jordens et al., 2001). Subsequently, cells were infected with *S. aureus* wt for 1 h and images of living cells were analyzed (Figure 2D). We observed colocalization of Rab7 and RILP in Safs membranes, suggesting that Rab7 is interacting with RILP to promote Saf elongation. Interestingly, we observed inhibition of Saf elongation when RILPΔN was used. Taken together these findings indicate that *S. aureus* uses the MT-motor kinesin-1 and the protein RILP of the host cell to promote Saf elongation.

### Rab1b is recruited to *S. aureus*-induced filaments

**Figure 3:**
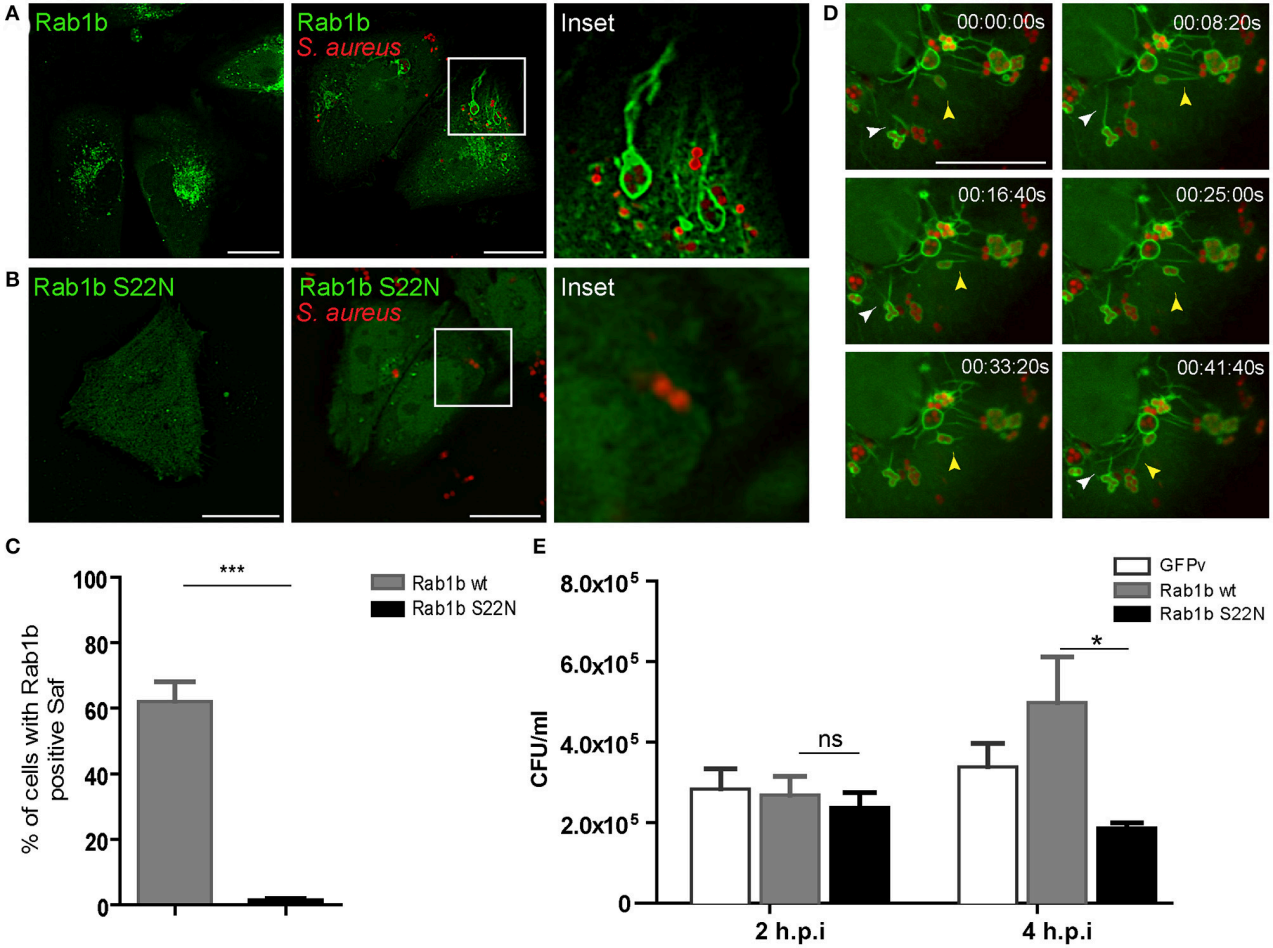
Rab1b is recruited to *S. aureus*-containing phagosomes and Safs. **(A)** CHO cells stably overexpressing GFP-Rab1b were infected with *S. aureus* wt for 1 h. Afterwards non-internalized bacteria were removed by washing and living cells were observed by confocal microscopy. **(B)** CHO GFP-Rab1b S22N transiently transfected cells were infected for 1 h with *S. aureus* wt. Bacteria were labeled with rhodamine red. **(C)** The total percentage of infected cells that generate Rab1b-positive Safs was determined in cells overexpressing Rab1b wt (gray bar) or Rab1b S22N (black bar). Data is representative of three independent experiments. **(D)** Images show CHO living cells stably overexpressing GFP-Rab1b infected with *S. aureus* for 1 h and visualized by confocal microscopy. White arrowhead indicates Saf branching and extension. Yellow arrowhead shows a Rab1b-positive phagosome with Saf that extends toward the cell periphery and finally reaches other bacteria-containing phagosome. Details of representative Saf phenotypes are shown, and the relative time-points are expressed as h:min:s. **(E)** The graph shows the quantification of the number of CFU/ml in CHO cells transfected with GFP vector alone (control, white bars), GFP-Rab1b (gray bars) or GFP-Rab1b S22N (black bars) and infected with *S. aureus* wt for 2 or 4 h. In all cases data show mean ± SEM of three independent experiments (*n* = 150 cells/condition). ^*^P ≤ 0.01; ^***^P ≤ 0.0001 from two-tailed Student *t*-test. Scale bar: 20 μm.

Next we were interested in determining the nature of the tubular-like structures using different sub-cellular markers. To determine whether *S. aureus*-filaments are enriched in endosomal associated membrane proteins, CHO cells were transiently transfected with different Rab proteins (Rab1b, Rab5, Rab7, Rab11, Rab24, Rab29, Rab32, or Rab34) and infected with *S. aureus* wt. Living cells were analyzed after 1 h post-infection by fluorescence microscopy. Rab24 and Rab29 were not recruited to *S. aureus-*containing vacuoles neither to Safs whereas Rab5, Rab11, Rab32, and Rab34 were recruited to *S. aureus* phagosome but not to Saf membranes (Figure S2). To our surprise, as depicted in Figure 3A *S. aureus* phagosomes were decorated with Rab1b and they presented numerous tubular structures also marked by the protein in approximately 60% of the infected cells (Figure 3C). Analysis of video microscopy showed dynamic tubules marked with Rab1b emerging form *S. aureus* phagosomes that were able to branch, contract, elongate and reach other bacteria-containing phagosomes (Figure 3D and Movie S2). In order to study the participation of a functional Rab1b in Saf generation and elongation, CHO cells were transfected with EGFP-Rab1b S22N (a dominant negative mutant locked in a GDP-bound conformation) and infected with *S. aureus* wt. As show in Figure 3B cells overexpressing Rab1b S22N did not present Rab1b positive Safs after 1 h post-infection. Next, we analyzed the importance of Rab1b in *S. aureus* replication. Therefore, CHO cells overexpressing GFP-Rab1b wt or GFP-Rab1b S22N were infected with *S. aureus* for 2 or 4 h and CFU were determined. As shown in Figure 3E, cells overexpressing Rab1b S22N presented a significant decrease in bacterial replication compared to cells overexpressing Rab1b wt. These results indicate that Rab1b is present in Saf membranes and that the activity of this Rab GTPase is necessary for Saf formation and efficient bacteria replication.

### Saf membranes are decorated with the autophagic protein LC3

**Figure 4:**
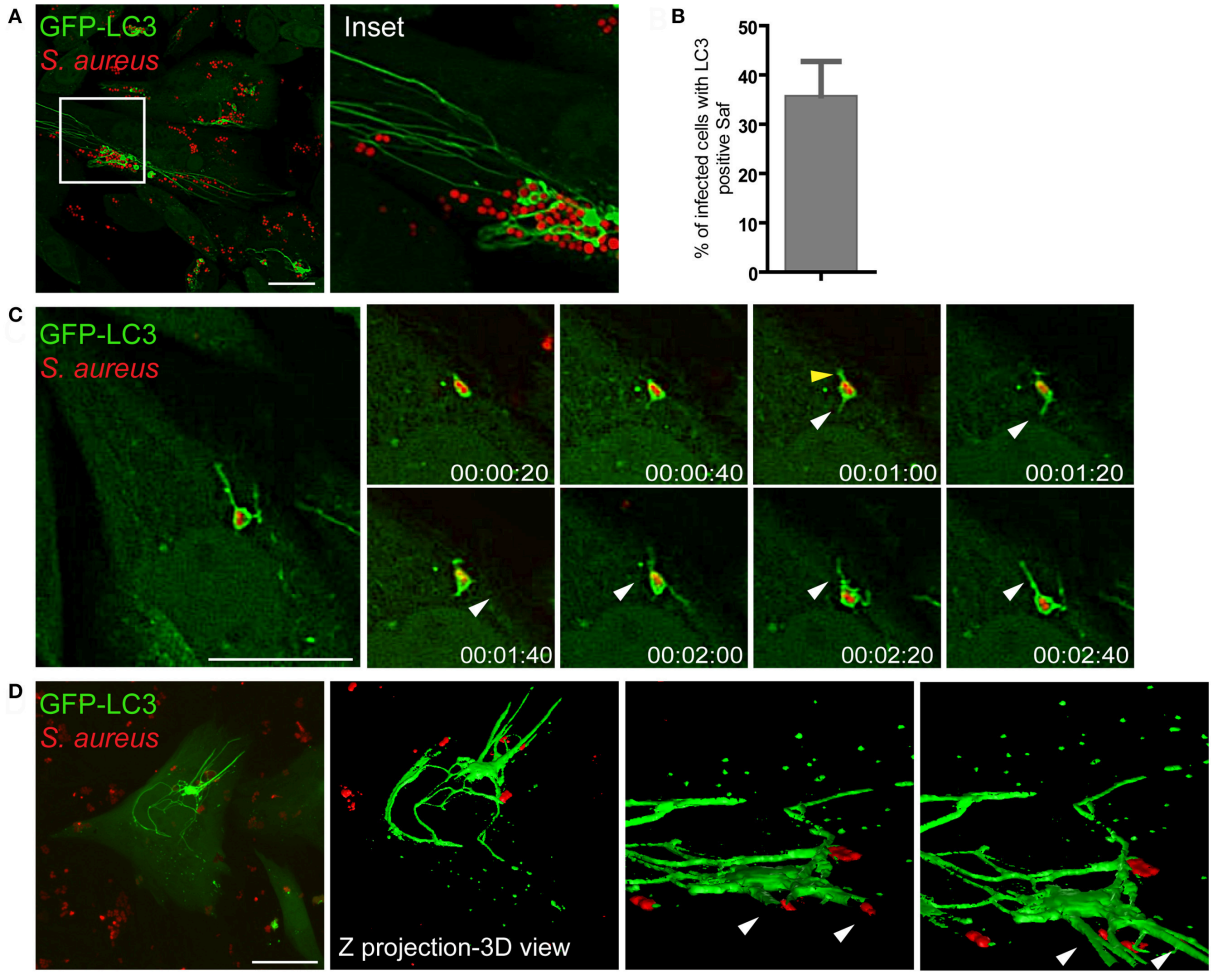
Saf membranes are decorated by the autophagic protein LC3. **(A)** CHO cells stably expressing GFP-LC3 were infected with rhodamine red labeled *S. aureus* for 1 h. **(B)** Quantification of the percentage of infected cells that presented Saf formation. Data show mean ± SEM of three independent experiments (*n* = 150 cells/condition). **(C)** Images show CHO living cells stably overexpressing GFP-LC3 infected with *S. aureus* for 1 h visualized by confocal microscopy. White arrowheads indicates a Saf emerging from the *S. aureus*-containing phagosome toward the perinuclear region and yellow arrowheads indicate Saf emerging toward the opposite direction. Relative time-points are expressed as h:min:s. **(D)** CHO cells stably expressing GFP-LC3 were infected with *S. aureus* wt for 1 h. Z stack images were processed using Image J to construct a 3D image. Bacteria were labeled with rhodamine-red. Scale bar: 20 μm.

We have previously demonstrated that Rab7 is a critical protein involved in the maturation of autophagic vacuoles and required for the progression of the pathway (Gutierrez et al., 2004). In addition, we have also shown that the small GTPase Rab1b is involved in the formation of autophagosomes (Zoppino et al., 2010). Therefore, we were next interested in determining whether the filamentous structures generated by *S. aureus* were related to the autophagy pathway by assessing the autophagic protein LC3. To analyze the presence of LC3 in Saf membranes CHO cells stably overexpressing GFP-LC3 were infected with *S. aureus* wt for 1 h and living cells were observed by confocal microscopy. As shown in Figure 4A we noted the formation of tubular GFP-LC3 positive structures extending from *S. aureus* autophagosomes (Movie S3). Time-lapse series show the extension of tubular membrane emerging form the autophagosome that contains *S. aureus* (Figure 4B). Quantification analysis indicates that approximately 35% of the infected cells showed one or more Safs marked with LC3 after 2 h.p.i. (Figure 4C). In order to analyze in more detail Safs structure, CHO GFP-LC3 cells were infected with *S. aureus* wt for 1 h and Z stacks were acquired with a confocal microscopy, images were processed with the ImageJ program, and a three dimensional reconstruction was performed (Figure 4D).

**Figure 5:**
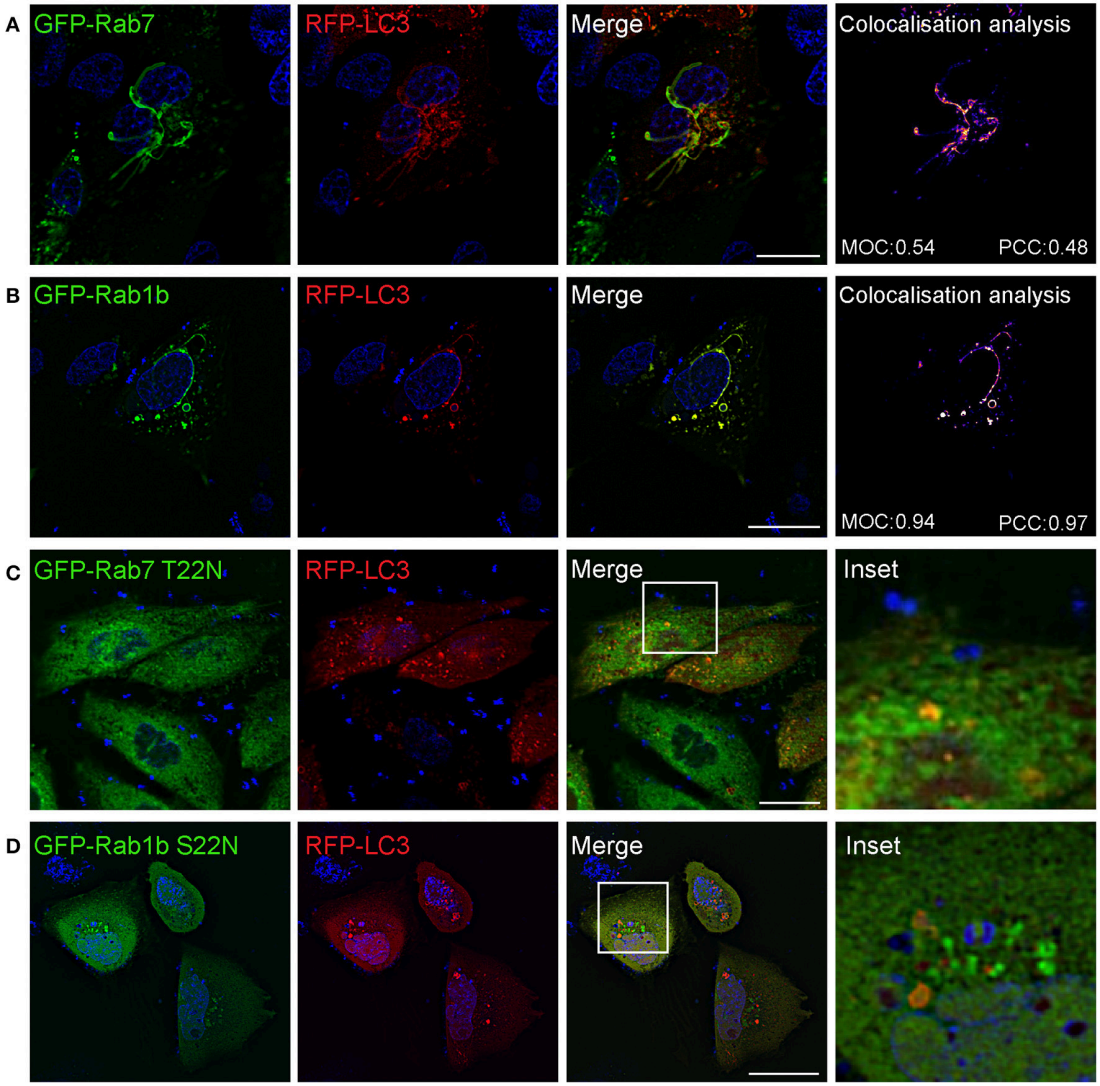
**Saf formation requires active Rab7 and Ra1b GTPases**. **(A)** CHO cells stably expressing GFP-Rab7 were transfected with RFP-LC3 and infected with *S. aureus* wt for 1 h. **(B)** CHO cells were co-transfected with GFP-Rab1b and RFP-LC3 and subsequently infected with *S. aureus* wt for 1 h. Colocalisation analysis of image A and B was performed using Image J to determine Mander's Overlap Coefficient (MOC) and Pearson Correlation Coefficient (PCC). **(C)** CHO cells stably expressing GFP-Rab7 T22N were transfected with RFP-LC3 and infected with *S. aureus* wt for 1 h. **(D)** CHO cells were co-transfected with GFP-Rab1b S22N and RFP-LC3 and subsequently infected with *S. aureus* wt. In all cases **(A–D)** bacteria were labelled with Hoechst and living cells were visualized by confocal microscopy. Scale bar: 20 μm. Images are representative of two different experiments.

We next studied if Safs marked with LC3 corresponded to the same structures marked with Rab7 or Rab1b. For this purpose, CHO cells stably overexpressing EGFP-Rab7 were transfected with RFP-LC3 and infected with *S. aureus* wt for 1 h. Rab7 and LC3 showed a colocalization of 50% approximately, indicating that both proteins are recruited to the same Saf membrane. When cells were co-transfected with GFP-Rab1b and RFP-LC3, and subsequently infected with *S. aureus* wt for 1 h, we also observed a high degree of colocalization (90% approximately) of both proteins at the Saf membranes (Figures 5A,B). In contrast, in cells co-expressing LC3 and Rab7 T22N infected with *S. aureus* wt for 1 h no tubules labeled with LC3 were observed reflecting an impaired Saf biogenesis (Figure 5C). A similar effect was observed when CHO cells were cotransfected with RFP-LC3 and GFP Rab1b S22N and infected with *S. aureus* wt for 1 h (Figure 5D). Taken together, these results describe a particular phenotype induced by intracellular *S. aureus* with the formation of tubular membrane structures that recruit Rab1b, Rab7, and LC3 proteins and that depends on active conformation of the GTPases Rab1b and Rab7.

### An *S. aureus* secreted factor is necessary for saf formation

**Figure 6:**
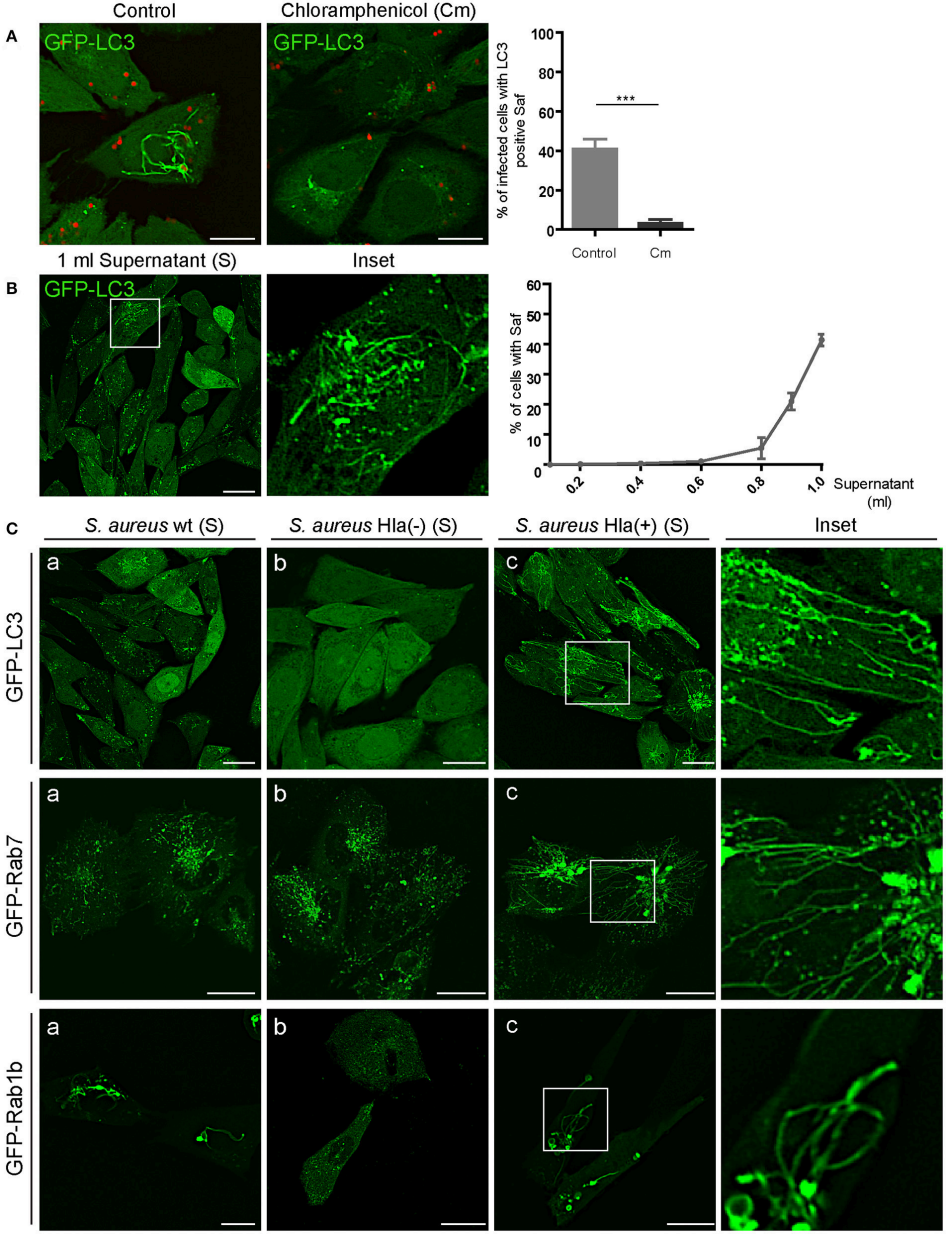
*S. aureus* culture supernatant induces tubular structures formation. **(A)** Left panel: CHO GFP-LC3 stably transfected cells were infected with *S. aureus* wt for 1 h. Right panel: CHO GFP-LC3 stably transfected cells were infected with *S. aureus* wt that was previously treated with chloramphenicol (Cm). Bacteria were labeled with rhodamine red. The graph shows the percentage of cells with Saf formation. **(B)** CHO cells stably expressing GFP-LC3 were incubated with increasing volumes (ml) of *S. aureus* wt supernatant (free of bacteria). The graph shows the percentage of cells with Saf formation. **(C)** Panels a-c show cells treated with the supernatant obtained from the *S. aureus* strains wt, Hla (–) and Hla (–) + p.Hla, respectively. Upper panel shows CHO GFP-LC3 stably transfected cells; middle panel CHO cells stably expressing GFP-Rab7 and lower panel CHO cells transfected with GFP-Rab1b. Live imaging was performed in all conditions by confocal microscopy. In all cases data show mean ± SEM of two independent experiments (*n* = 100 cells/condition). ^***^P ≤ 0.0001 from two-tailed Student *t*-test. Scale bar: 20 μm

*Staphylococcus aureus* produces an extensive variety of exoproteins that are secreted in the last exponential phase of growth in a culture media (Dinges et al., 2000). In order to determine if the formation of tubular Safs is induced by an *S. aureus* secreted factor, we treated *S. aureus* with chloramphenicol (Cm), a bacteriostatic antibiotic that inhibits protein synthesis. As depicted in Figure 6A, in CHO cells stably expressing GFP-LC3 that were infected with *S. aureus* pre-treated with Cm, Saf formation was drastically reduced (only 7% of the cells presented tubules) compared to the control condition (40%). To corroborate that Saf formation is induced by a *S. aureus* secreted protein we treated CHO GFP-LC3 cells with different concentrations of filtered supernatant of a *S. aureus* culture (Figure 6B). Interestingly, cells treated with increasing concentrations of the supernatant (S) of *S. aureus* showed Saf formation after 30 min of incubation. We performed a dose curve of undiluted supernatant of a protein concentration of 34 μg/ml final volume and determined that 0.1 ml was sufficient to induce Saf formation (Figure 6B). As a control, sterile culture media was added to CHO cells in the same concentration and no Safs were observed (Figure S3A). Hence, we next tested the culture supernatant of three *S. aureus* strains: wild type, Hla (–) (a mutant deficient for α-toxin), and Hla (+) (a mutant complemented with the α-toxin-encoded plasmid). Strikingly, we observed that in the cells treated with the supernatant of the Hla (–) strain no Safs were visualized, in contrast the strains that produce α-toxin did induce tubular structures marked with LC3 (Figure 6C). To determine if this effect was also observed in cells overexpressing Rab7 or Rab1b, CHO cells stably expressing GFP-Rab7 or cells transfected with EGFP-Rab1b were treated with the supernatant obtained from the three strains: wt, Hla (–) and Hla (+) and we observed that in concordance with the results obtained above, Hla (–) supernatant did not induced Saf formation marked with Rab7 neither Rab1b. These findings strongly suggest that the α-hemolysin is the *S. aureus* protein responsible for Saf formation.

### The α-hemolysin induced filaments are inhibited by cAMP

**Figure 7:**
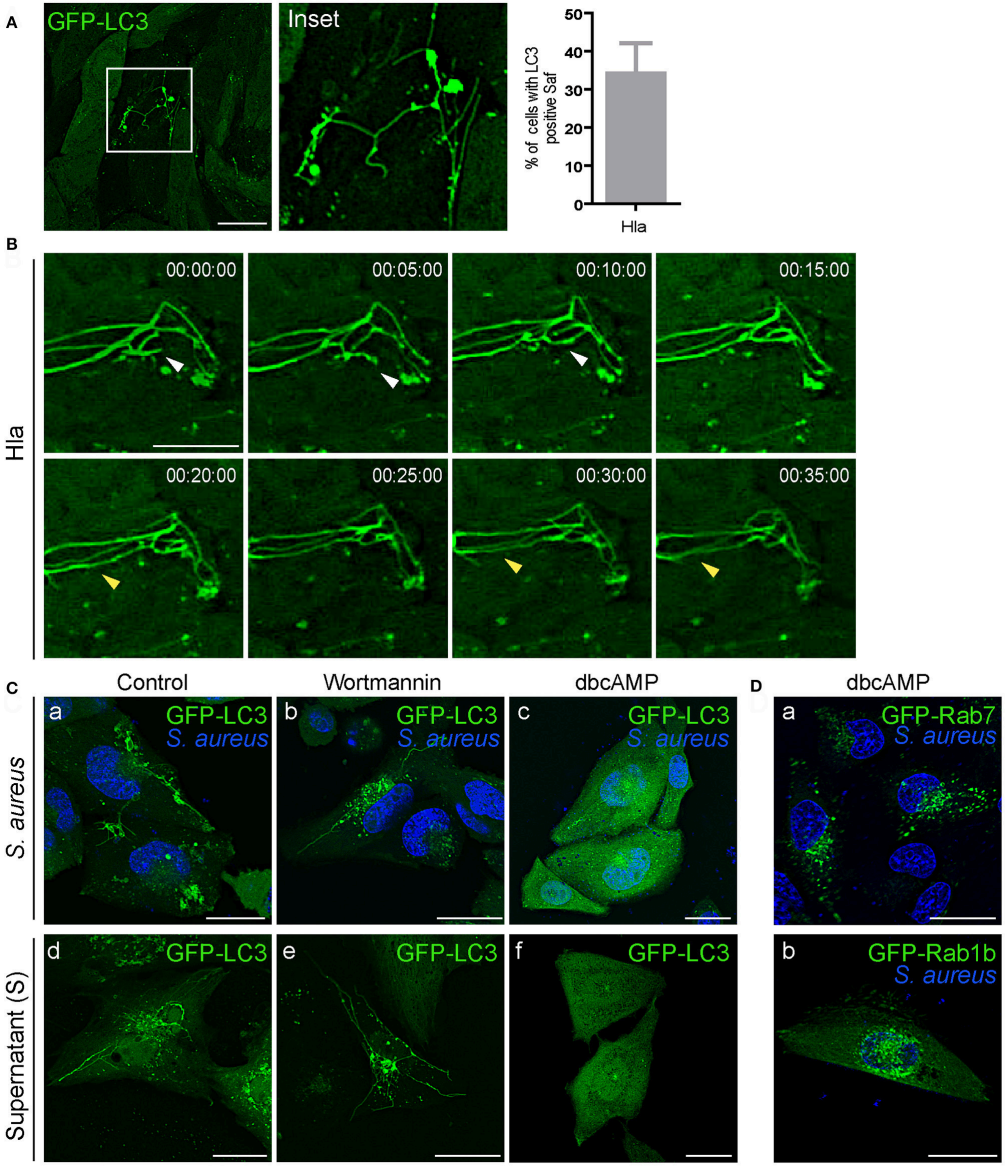
α-hemolysin induces Saf formation that is hampered by increasing the cAMP levels. **(A)** CHO GFP-LC3 stably transfected cells treated with 10 μg/ml of alpha hemolysin (Hla) of *S. aureus*. The graph shows quantification of percentage of cells treated with Hla that show Saf formation. **(B)** Movie depicting CHO cells overexpressing GFP-LC3 treated with 10 μg/ml of alpha hemolysin (Hla). White arrowhead shows Saf extension and subsequent fusion. Yellow arrowhead indicates parallel Safs that contact. Relative time-points are expressed as h:min:s. **(C)** Upper Panel: CHO cells stably expressing GFP-LC3 were infected with *S. aureus* wt for 1 h (a). CHO GFP-LC3 were infected with *S. aureus* wt for 30 min and then wortmannin was added (b) or cells were treated with dbcAMP for 30 min and then infected (c). Lower Panel: CHO GFP-LC3 cells were incubated with *S. aureus* supernatant (d) or pre-treated with wortmannin (e) or dbcAMP (f) for 30 min before adding the *S. aureus* supernatant. **(D)** CHO GFP Rab7 stable cells (a) or CHO cells overexpressing GFP-Rab1b (b) were treated with dbcAMP for 30 min and then infected with *S. aureus* wt for 1 h. In all cases living cells were observed by confocal microscopy.

We have previously demonstrated that Hla secretion is necessary for autophagy activation and the process occurs through a PI3K/Beclin1-independent mechanism (Mestre et al., 2010). To assess the role of Hla in Saf formation, we tested the effect of the purified α-hemolysin in CHO cells stably expressing GFP-LC3 (Figure 7A). For this purpose, cells were treated with 10 μg/ml of the toxin for 1 h (Mestre et al., 2010) and living cells were analyzed by confocal microscopy. As depicted in Figure 7A, 35% of cells show tubular structures decorated with LC3 protein just by the addition of the purified toxin. Analysis of time laps series show dynamic Safs marked with LC3 induced by α-hemolysin (Figure 7B). To rule out that Saf formation could be an effect of the activation of the autophagy pathway CHO GFP-LC3 cells were incubated with starvation media and no Safs were visualized (Figure S3A). It has been previously shown that alpha hemolysin is recognized, in epithelial cells, by it superficial receptor ADAM10 (Wilke and Bubeck Wardenburg, 2010). Indeed, ADAM10 is internalized by clathrin-dependent endocytosis specifically interacting with AP2 in neuronal cells (Marcello et al., 2013). Taking these findings into account, we next analyzed if the induction of Saf formation by Hla is affected when clathrin-dependent endocytosis is inhibited. Thus, we treated CHO cells stably expressing GFP-LC3 with dynasore, a GTPase inhibitor that targets dynamin (Macia et al., 2006) or PitStop 2, a clathrin terminal domain (TD) inhibitor (von Kleist et al., 2011) and then added purified Hla. As shown in Figure S3B no Saf formation was observed when cells were treated with the inhibitors. This result clearly indicates Hla induces tubular compartments only when is endocytosed by a clathrin-dependent process.

Previously we have reported that cAMP and the effector proteins, EPAC (exchange protein activated by cAMP) and Rap2b, inhibit the autophagic response induced by α-hemolysin (Mestre and Colombo, 2012). Hence, we next assessed the effect of wortmannin (PI3K inhibitor) and dbcAMP (a membrane permeable cAMP analog) on the formation of Safs (Figure 7C). As expected wortmannin had no effect, on the contrary, dbcAMP inhibited the formation of *S. aureus* filaments induced by the bacteria or by the added supernatant. Thus, the inhibition of the autophagic response generated by Hla seems to be sufficient to inhibit the formation of LC3 tubular structures. In addition, we analyzed the effect of dbcAMP on Safs labeled with Rab7 or Rab1b, for this purpose CHO cells stably expressing GFP-Rab7 or GFP-Rab1b were treated with 1 mM of dbcAMP for 30 min and then infected with *S. aureus* wt. After 1 h post-infection living cells were analyzed by confocal microscopy and no Safs were visualized (Figure 7D). These findings strongly indicate that cAMP is a key regulator of the *S. aureus* induced filaments and that they are related to the autophagy pathway induced by the toxin.

**Figure 8:**
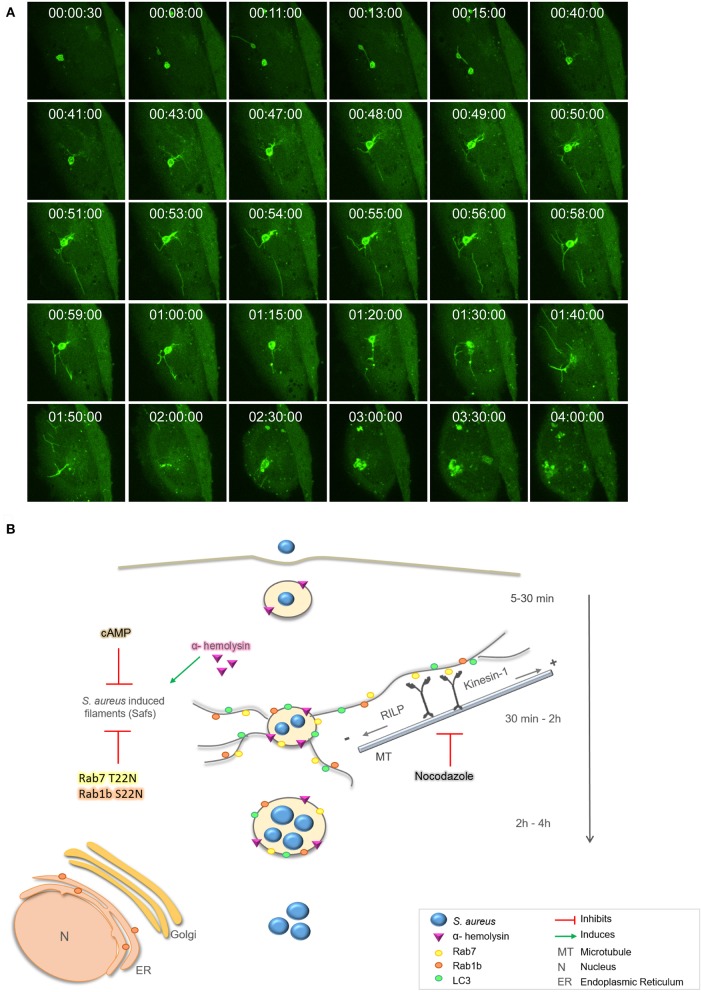
Proposed model for intracellular *S. aureus* tubular formation. **(A)** CHO GFP-LC3 cells were infected with *S. aureus* wt for 30 min, then, extracellular bacteria were washed. One cell, was analyzed by spinning disk microscopy during 4 h post-infection. Relative time-points are expressed as h:min:s. **(B)** Proposed model: After 30 min of infection *S. aureus* recruits Rab7, Rab1b, and LC3 proteins to the containing phagosome and Saf formation is induced. Rab7 interacts with RILP and Kinesin-1 to promote membrane elongation via microtubules. *S. aureus* α-hemolysin induces Saf formation whereas nocodazole, cAMP, or overexpression of the dominant negative mutants Rab1b S22N and Rab7 T22N negatively regulate Saf formation and bacteria growth.

Next, we performed live cell fluorescence microscopy using a spinning disk microscope to follow living cells infected with *S. aureus* for prolonged periods of time (i.e., up to 4 h). CHO GFP-LC3 cells were infected with *S. aureus* wt for 30 min [indicated as time point 0 (00:00:00 s)], to allow bacteria internalization, and then cells were plated in a specific chamber at 37°C with 5% of CO_2._ Images were acquired every 30 s during 4 h. As depicted in Figure 8A, at initial times we observed LC3 recruited to *S. aureus* phagosomes and after 10 min approximately we visualized Saf formation emerging from the phagosome. This phagosome after 15 min showed another Saf that contacted a neighbor phagosome. As visualized on Figure 8A and Movie S4 after 40 min more Safs were visualized extending in different directions and continued until 1 h and 50 min of infection approximately. The *S. aureus* containing-phagosome was observed during the next 2 h and no Saf formation was detected until the end of the movie (4 h).

*Staphylococcus aureus* scape from the phagosome depends on bacterial virulence factors, such as toxins, and it has been described to occur between 3 and 6 h post-infection (Giese et al., 2011; Münzenmayer et al., 2016). However, there is a discrepancy whether the bacteria only replicates in the cytoplasm (Grosz et al., 2014) or in the phagosomal compartment (Schröder et al., 2006; Kubica et al., 2008; Flannagan et al., 2016). Here we have observed bacteria replication within the phagosomal compartment that shows Saf formation at early infection times (Figure 8). Taken together these results describe a particular intracellular phenotype induced by *S. aureus* characterized by the formation of dynamic tubular membrane structures labeled with Rab1b, Rab7, and LC3 proteins that occur at early infection times (30 min–2 h post-infection) which are induced by α-hemolysin and regulated by cAMP levels.

## Discussion

In previous studies we have demonstrated that *S. aureus* induces autophagy activation in infected cells, generating LC3-positive vesicles (Mestre et al., 2010). These results are consistent with previous studies showing that *S. aureus* transits via autophagosomal compartments colocalizing with LC3 (Schnaith et al., 2007). These authors postulate that the autophagosomes provide a protective niche for intracellular bacteria, allowing replication and then, bacteria escape into the cytoplasm finally inducing cell death through apoptosis. In addition, Koide and collaborators have previously shown that S*. aureus* recruits in a high percentage the GTPase Rab7 to the phagosomal membrane (Seto et al., 2011).

In the present study we have analyzed S. *aureus* infection in living cells displaying the formation of highly dynamic membrane filaments (Figure 8B). These findings are in concordance with a recent report that shows LC3 positive membranes surrounding *S. aureus* phagosome (Neumann et al., 2016). Here we have shown that *S. aureus* is able to induce tubular structures that emerge from the pathogen-containing phagosome. These filaments have autophagic characteristics, evidenced by the presence of LC3, Rab7, and Rab1b, proteins involved in the autophagic pathway (Zoppino et al., 2010; Amaya et al., 2015). Indeed, our results clearly indicate that Safs biogenesis are Rab1b and Rab7-dependent, as no LC3-tubular structures were visualized when cells were cotransfected with a Rab1b S22N or Rab7 T22N (dominant negative mutants). These findings indicate that *S. aureus* needs a functional Rab1b and Rab7 to induce membrane elongation and Saf formation (Figure 8B). A plausible explanation for these observations is that Rab7 exert this function by, interacting with motor proteins involved in membrane trafficking (Guerra and Bucci, 2016). In concordance with these results we also presented evidence that an intact microtubule cytoskeleton is necessary for proper Saf formation. Microtubules and the motor proteins, kinesin and dynein, are essential for correct transport of vesicles (Bananis et al., 2004). Kinesins use microtubules to transport cargo toward the cell periphery; conventional kinesin (Kinesin-1 or Kif5B) is involved in early/late endosome transport (Hirokawa et al., 2009). Dyneins use microtubules to drive minus end directed retrograde transport, and the Rab7-interacting lysosomal protein (RILP) was found to interact with the dynein-dynactin motor complex (Jordens et al., 2001). Hence, we have demonstrated that Kif5 and RILP are essential for Saf elongation; thus, it is likely that via the association with Rab7 these proteins facilitate the extension of the phagosomal membranes through microtubules. Both forces, toward the periphery and the cell center are necessary to elongate Saf membrane (Figure 8B). Other pathogens also regulate motor proteins in order to control intracellular transport and establish a replicative niche. For example, PipB2 a *Salmonella* protein which localizes to the *Salmonella*-containing vacuole, is associated with tubular-vesicular structures at the cell periphery (Knodler and Steele-Mortimer, 2005). PipB2 is a specific adaptor for Kinesin-1 that regulates the position of late endosomal compartments in a microtubule dependent manner (Leone and Méresse, 2018). We believe that *S. aureus* similar to *Salmonella* interacts with motor proteins to control vesicular trafficking and membrane elongation allowing Saf formation induced by alpha toxin. Future studies will be designed to address this point. An important issue is that the *Salmonella-*induced filaments or SIF have been described as long tubular membrane compartments extending from the *Salmonella*-containing vacuole (SCV) (Garcia-del Portillo et al., 1993). These structures are characterized by the presence of specific protein markers, such as Rab7, LAMP-1 and other lysosomal markers (Garcia-del Portillo et al., 1993; Drecktrah et al., 2008; Rajashekar et al., 2008). Furthermore, Hensel and collaborators have recently demonstrated that SIFs are not related to autophagosomes as they did not recruited LC3 to the membrane (Krieger et al., 2014). Taken together these features clearly indicate that the tubular filaments of *Salmonella* (SIF) are different from those induced by *S. aureus* (Saf).

We have previously reported that Rab1b, a regulatory protein that is required for the ER-to-Golgi traffic at a pre-Golgi stage, participates in autophagosome formation. Rab1b wt colocalized with LC3, either endogenous or overexpressed, but when Rab1b dominant negative mutant or a siRNA to deplete this Rab was used a marked reduction in the formation of autophagosomes was observed (Zoppino et al., 2010). Indeed, our current results demonstrate that Rab1b is present in Saf membranes and when the dominant negative mutant was overexpressed no Saf formation was observed, indicating that Rab1b is involved in *S. aureus* filaments formation probably by its functional role in the autophagy pathway. Interestingly when a CFU assay was performed bacteria replication was markedly reduced in Rab1b S22N and Rab7 T22N cells, suggesting that Saf formation is important for proper bacterial growth and that Rab1b and Rab7 have a key role in *S. aureus* life cycle. A plausible explanation is that Safs are providing membranes and likely others components necessary for the maintenance of the *S. aureus* vacuole. Similar to our results, a recent publication indicates that depletion of Rab1b in *Salmonella* infected cells leads to more cytoplasmic bacteria replication and less permanence in vacuoles, compared to control conditions (Huang et al., 2011).

In the present report we have also demonstrated that *S. aureus* supernatant, which contains bacterial exoproteins, is capable of inducing Saf formation. Interestingly, when the supernatant of a *S. aureus* strain incapable of producing α-hemolysin (Hla) was used no Safs were observed, indicating that the toxin is necessary to induce Safs. Indeed, we showed that the purified toxin Hla was sufficient to produce Saf generation in CHO cells stably overexpressing GFP-LC3 (Figure 8B). In a previous publication we demonstrated that Hla is the *S. aureus* secreted factor responsible for autophagy induction in infected cells and also in cells treated with the purified toxin (Mestre et al., 2010). Furthermore, we previously reported that the autophagic response induced by α-hemolysin is PI3K/Beclin-1 independent and that cAMP has a key role in its regulation. Hence, cAMP is able to inhibit the autophagic response induced by the toxin, indicating that the mechanism of regulation is through a non-canonical autophagy (Mestre and Colombo, 2012). In addition, in the present report we have demonstrated that cAMP negatively regulates Saf biogenesis. It is likely that Hla is inducing Saf formation as a consequence of the activation of the autophagic pathway; however, more experiments will be needed to fully elucidate the mechanism at the molecular level.

Interestingly, *S. aureus* replication significantly decreased when Safs were inhibited by adding nocodazole, an agent that depolymerizes microtubules, to infected cells. The same effect was observed by the overexpression of Rab7 and Rab1b dominant negative mutants. Thus, these findings clearly indicated that *S. aureus* has the ability to induce tubular structures that are necessary for its proper replication. Saf formation may play a key role in *S. aureus* replication probably by providing membrane for the replicative niche.

In summary, our study demonstrates that *S. aureus* induces striking tubular structures that protrude from the bacteria containing phagosome, and also branches and contact other phagosomes in a microtubule- dependent manner. Importantly, these tubules are necessary for *S. aureus* replication and the exotoxin α-hemolysin is essential for *S. aureus* filaments (Saf) formation. Thus, we believe that the present results contribute to our understanding of the intracellular survival and the importance of Hla in this pathogen infection.

## Author contributions

ML and MG Methodology and Investigation. ML and MC Writing, Review and Editing. MC Funding Acquisitions, Resources and Supervision.

### Conflict of interest statement

The authors declare that the research was conducted in the absence of any commercial or financial relationships that could be construed as a potential conflict of interest. The reviewer RF and handling Editor declared their shared affiliation.
