# Supplementary material for: Staphylococcus aureus Alpha-Toxin Induces the Formation of Dynamic Tubules Labeled with LC3 within Host Cells in a Rab7 and Rab1b-Dependent Manner
Source: Front Cell Infect Microbiol. 2017 Oct 4;7:431. doi: 10.3389/fcimb.2017.00431 (PMC5632962; doi:10.3389/fcimb.2017.00431)
Supplement: Supplementary file 1 [file DataSheet1.pdf]

Supplementary Material

Staphylococcus aureus alpha-toxin induces the formation of dynamic tubules labeled with LC3 within host cells in a Rab7 and Rab1b-dependent manner.

María Milagros López de Armentia, María Celeste Gauron and María Isabel Colombo*

* **Correspondence**: Corresponding author: María I. Colombo.
E-mail: mcolombo@fcm.uncu.edu.ar

1. Supplementary Figures

Figure S1

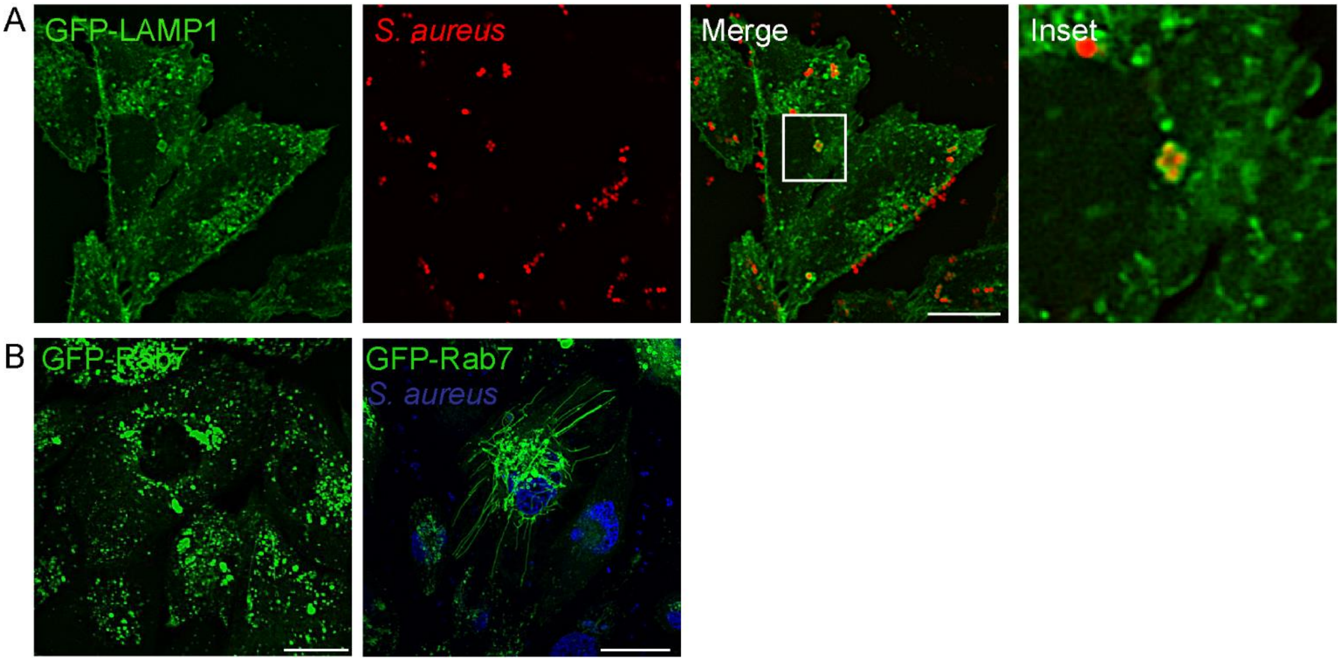

Figure S1. LAMP-1 is not recruited to *S. aureus* induced filaments (Safs) and Safs are specific of infected cells. (A) CHO cells were transfected with GPF LAMP-1 and then infected with *S. aureus* wt (Rhodamine Red) for 1 hour. **(B)** CHO GFP-Rab7 cells not infected (left panel) or infected with *S. aureus* wt for 1 hour (right panel). Living cells of both experiments were observed by confocal microscopy Scale Bar: 20 µm. Images are representative of two similar experiments.

Figure S2

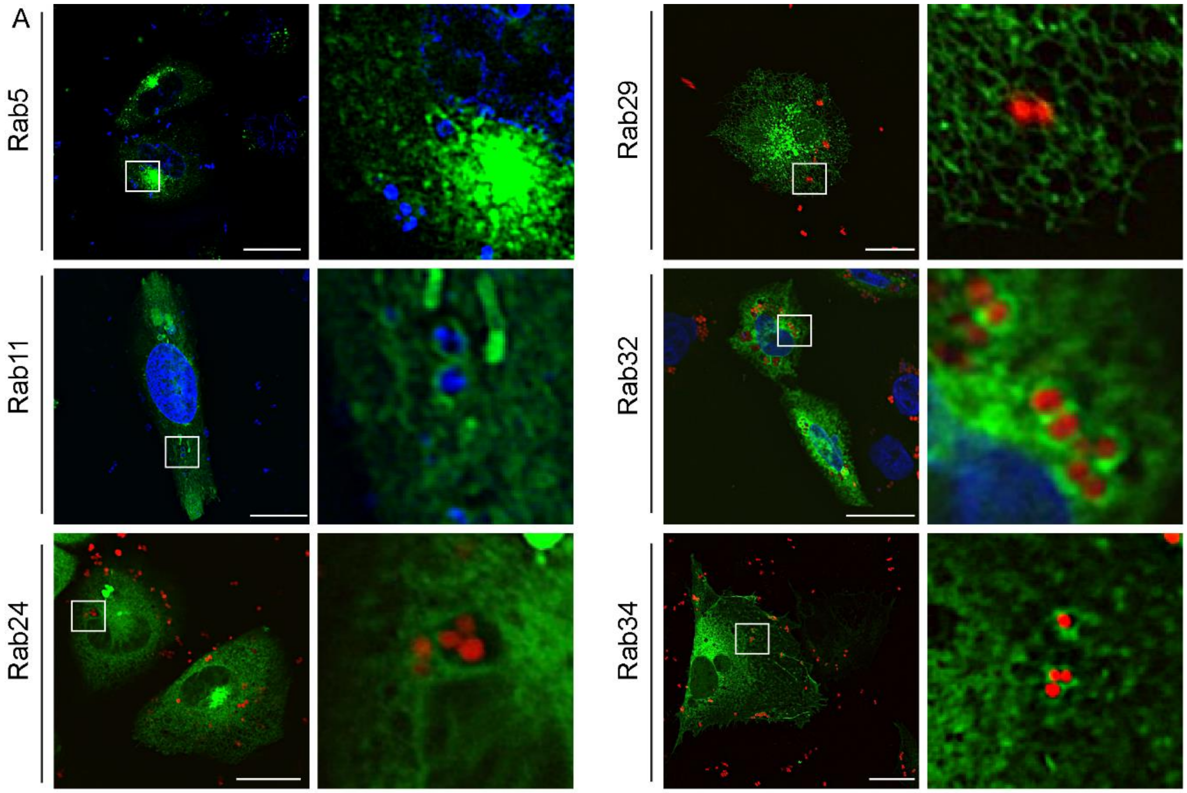

B

Rab Protein	*S. aureus* phagosome	*S. aureus* filaments (Saf)
Rab1b	+	+
Rab5	+	-
Rab7	+	+
Rab11	+	-
Rab24	-	-
Rab29	-	-
Rab32	+	-
Rab34	+	-

Figure S2. Localization of Rab GTPases on *S. aureus* phagosome and on Saf membrane. (A) CHO cells transfected with Rab GTPases plasmids and infected with *S. aureus* wt. The plasmids used were: EGFP-Rab5, EGP-Rab11, EGFP- Rab24, EGFP-Rab29, EYFP-Rab32 and EGFP-Rab34. Bacteria were labelled with rhodamine-red or Hoechst. All cases were visualized as living cells by confocal microscopy. Scale bar: 20 µm. **(B)** Table indicates Rab GTPases recruitment to *S. aureus* phagosome and/or to *S. aureus*-induced filaments.

Figure S3

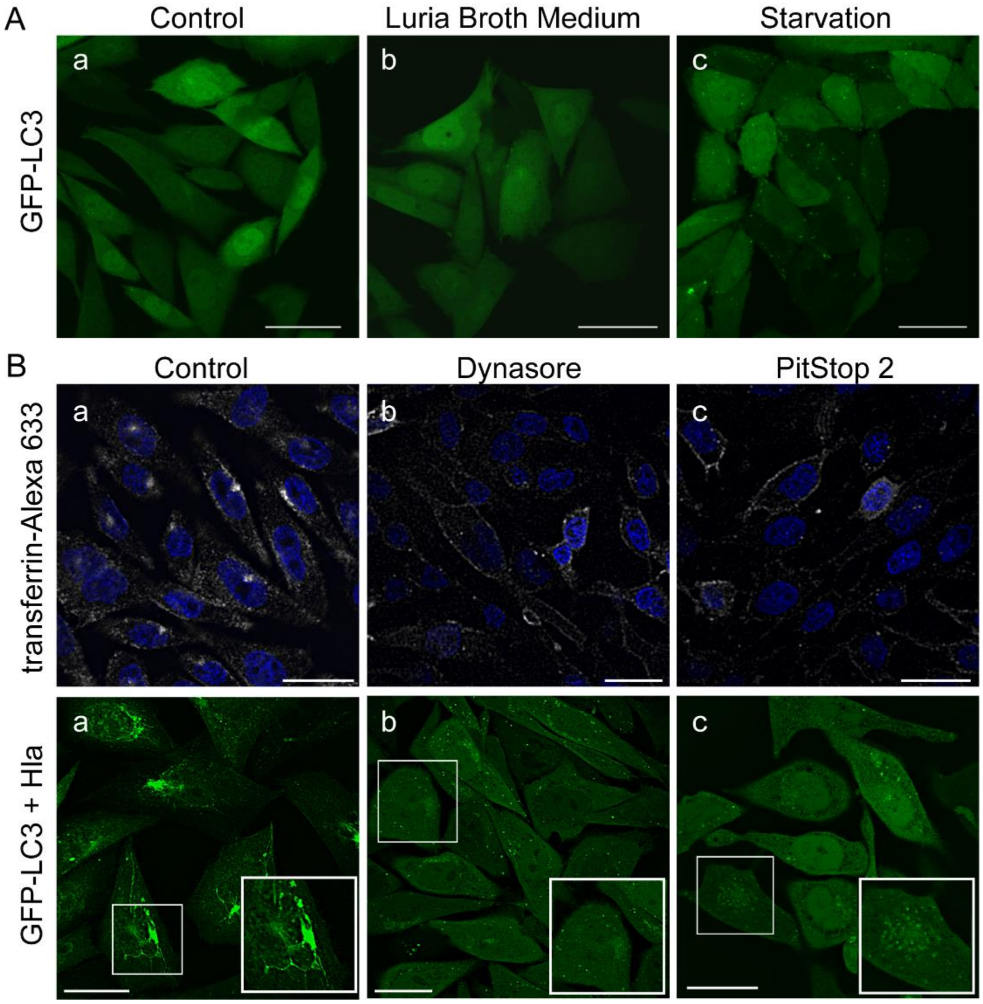

Figure S3. No Safs are visualized in CHO cells treated with LB or under starvation conditions and alpha hemolysin induces tubular structures only when is endocytosed by a clathrin-dependent process. (A) CHO GFP-LC3 cells were left untreated (control), incubated with bacterial culture media (Luria Broth) or treated with Hank´s solution for 2 hours (starvation) and living cells were analyzed by confocal microscopy. **(B)** Top panel: CHO cells were treated with 80 µm of dynasore (b) or 20 µm of PitStop2 (c) for 1 hour or left untreated (a). After that period transferrin alexa 633 was added to all conditions for 20 minutes and then cells were washed with 1.5 M citric acid PBS pH 5.5 and fixed. Bottom panel: CHO cells were treated with 80 µm of dynasore (b) or 20 µm of PitStop2 (c) for 1 hour or left untreated (a). After incubation with the inhibitors, 10 µg/ml of Hla was added to all conditions and after 1 hour cells were processed by video microscopy. Images are representative of two similar experiments performed. Scale bar 20 µm.

2. Supplementary Video Legend

Movie S1. Live cells infected with *S. aureus* showed tubular membrane filaments marked with Rab7. Time lapse series of CHO GFP-Rab7 cells infected with *S. aureus* wt

for 1 hour (time point consider as cero). Images were acquired every 30 seconds. Bacteria were labelled with Hoechst. Images are representative of three similar experiments performed.

Movie S2. Time lapse series showing Saf labelled with Rab1b. CHO cells stably expressing GFP-Rab1b were infected with *S. aureus* wt (Rhodamine Red). One cell, exhibiting Saf formation, was analyzed by time-lapse microscopy at 1 h.p.i. Each frame was taken every 30 seconds.

Movie S3. Dynamic of Saf formation on living cells overexpressing LC3. CHO cells stably transfected with GFP-LC3 were infected with *S. aureus* wt (Rhodamine Red) for 1 hour. Time lapse series of infected cells were taken every 30 seconds.

Movie S4. Spinning Disk microscopy showing the dynamics of the Safs. CHO cells stably GFP-LC3 were infected with *S. aureus* wt for 30 minutes, subsequently extracellular bacteria was washed (this was considered time cero). One chosen cell was analyzed during 4 hours to study Saf formation and *S. aureus* infection. Each frame was taken every 30 seconds.
